# Thermal fluctuations affect embryonic physiology in a tropical agamid lizard

**DOI:** 10.1242/jeb.252800

**Published:** 2026-08-11

**Authors:** Amanda Ben, Debarpita Das, Nubla BM, Maria Thaker

**Affiliations:** ^1^ Centre for Ecological Sciences, Indian Institute of Science, Bangalore, 560012, Karnataka, India; ^2^ Indian Institute of Science Education and Research (IISER) Thiruvananthapuram, Vithura, 695551, Kerala, India

**Keywords:** Developmental plasticity, Incubation, Temperature, Reptile, Embryonic heart rate

## Abstract

Climate change is increasing both mean temperatures and thermal fluctuations, posing novel challenges for ectotherms. Reptilian embryos are vulnerable because of their limited thermoregulation and lack of parental care. Tropical reptiles often face high nest temperatures and fluctuations in the wild, yet most incubation studies use constant temperatures that fail to reflect this thermal variation. We examined the effects of constant versus fluctuating temperatures on embryonic physiology, development and hatchling size in the Indian rock agama, *Psammophilus dorsalis*. Eggs were incubated at constant 25°C, 30°C or 35°C, or under fluctuating regimes F2 (25–30°C) and F3 (25–30–35°C). Constant 35°C conditions caused complete mortality, whereas the warmest fluctuating regime (F3) resulted in dampened embryonic heart rate trajectories, faster egg mass gain, shorter development, lower hatching success and smaller hatchlings. These findings highlight the costs of thermal load and the importance of using fluctuating regimes to assess climate vulnerability in tropical reptiles.

## Teaser

**Summary:** Fluctuating high temperatures markedly reduced hatching success and hatchling body mass in the tropical agamid lizard *Psammophilus dorsalis*.

## INTRODUCTION

Climate change is altering thermal environments by increasing mean temperatures and intensifying fluctuations and extreme events (IPCC, 2023). Ectotherms such as reptiles are particularly vulnerable because their physiology, behavior and life history depend on environmental temperature (Angilletta, 2009). Although thermal effects on adult reptiles are well studied, the embryonic stage remains comparatively understudied despite its critical role in development (Pottier et al., 2026). Reptilian embryos typically develop in nests with little or no parental care and are therefore exposed to prevailing environmental conditions (Telemeco et al., 2009). Unlike later life stages, embryos possess limited capacity for behavioral thermoregulation. In many species, particularly lizards with small eggs, embryos cannot effectively move within the egg to avoid unfavourable temperatures (Telemeco et al., 2016). Consequently, all temperature-sensitive processes, including organogenesis, growth and metabolic regulation occur under the thermal regime of the nest site. Natural nests often experience daily fluctuations, sometimes exceeding 10–20°C (Shine and Elphick, 2001). Although an increasing number of experimental studies have examined the effects of fluctuating incubation temperatures (Noble et al., 2018a; Raynal et al., 2022), the majority of early work relied on constant temperature regimes that do not capture the dynamic thermal conditions of natural nests.

Fluctuating developmental temperatures can produce phenotypic outcomes that differ from constant regimes with the same mean temperature (Noble et al., 2018a). Moderate fluctuations tend to confer modest benefits, such as slightly higher hatching success and shorter incubation periods at cooler mean temperatures, whereas the same degree of fluctuations become detrimental as mean temperatures increase (Raynal et al., 2022). Embryonic heart rate provides a non-invasive measure of developmental responses (Du et al., 2009, 2011) and increases in heart rate with incubation temperature reflect elevated metabolic rates and faster development (Hall and Warner, 2020; Hulbert et al., 2017). Consistent with expectations from developmental plasticity, variation in thermal regimes during embryonic development can also affect hatching success, developmental duration, hatchling morphology and performance (Noble et al., 2018b).

Despite advances in understanding thermal developmental plasticity in reptiles, most experimental research has focused on temperate or subtropical species from North America, Australia and East Asia (While et al., 2018). Tropical reptiles, which often experience nest temperatures near their upper thermal limits, remain underrepresented. Recent multi-population assessments demonstrate that embryos from low-latitude regions face greater risks from climate change. For instance, although tolerance to chronic heat exposure by *Takydromus* sp. lizard embryos remain relatively consistent across latitudes, tolerance to acute heat exposure is lower in tropical and subtropical populations (Ma et al., 2024). In India, eggs of *Calotes versicolor* achieved high hatching success at ambient nest temperatures with a brief daily exposure to 35°C, but constant exposure to 33°C or 35°C reduced survival and produced smaller hatchlings (Radder et al., 2002). Limited knowledge of natural nest thermal regimes and embryonic responses to thermal variability in tropical Indian reptiles, however, constrains predictions of how rising temperatures and more frequent thermal extremes will affect reproductive outcomes.

In this study we investigated the effects of increased and variable incubation temperatures on embryonic physiology and development in *Psammophilus dorsalis*, the Indian rock agama, a widely distributed endemic lizard in India. Females lay eggs in shallow soil nests (∼8 cm deep), where embryos experience daily thermal variation (Balakrishna and Achari, 2014). Eggs were incubated under three constant temperatures (25°C, 30°C and 35°C) and two fluctuating regimes, F2 (alternated daily between 25°C and 30°C) and F3 (alternated daily among 25°C, 30°C and 35°C). Although these fluctuating treatments do not replicate the continuous diel temperature cycles experienced in natural nests, we aim to disentangle the effects of mean temperature, thermal variation and repeated exposure to elevated temperatures on embryonic development. Embryonic heart rate and egg mass were monitored non-invasively throughout development. Incubation duration, hatching success and hatchling size [snout to vent length, (SVL) and body mass] were measured. We predicted that warmer and more variable thermal regimes would increase embryonic heart rates and developmental rates while reducing hatching success and hatchling body mass, owing to elevated metabolic demands and potential oxygen limitation (Hall and Warner, 2020). By examining the effects of rising and varying incubation temperatures in an Indian agamid lizard, our study addresses a major geographical gap in reptile thermal biology and improves our understanding of how tropical reptiles may respond to climate warming.

## MATERIALS AND METHODS

### Study species and egg collection

*Psammophilus dorsalis* is a lizard species found naturally on rocky outcrops across peninsular India. Gravid females were captured by lassoing during the breeding seasons (April–September 2024 and 2025) from sites in and around Bangalore, Karnataka, India. Stage of gravidity was identified by gentle abdominal palpation and only females with calcified eggs were collected. Captured individuals were transported within 8 h in insulated boxes lined with ice packs to maintain moderate temperatures during transport. In the laboratory, gravid females were housed individually in glass terraria (60×30×25 cm) with tissue lining in the base, rocks as perches and a PVC pipe for refuge. The housing room was maintained at 25–27°C, which is within the preferred temperature range for females of *P. dorsalis* and was exposed to a 12 h photoperiod (07:00–19:00 h daylight). Incandescent lights (60 W) above each tank were turned on thrice a day for 30 min to provide basking opportunities. Mealworms and water were provided *ad libitum*. Terraria were also provided with a box filled with soil for oviposition, and females were monitored at 2 h intervals until eggs were laid. Once oviposition occurred (2–8 weeks post capture), eggs were collected and placed in plastic containers filled with moist vermiculite. Eggs from each clutch were distributed evenly across incubation treatments to control for potential maternal effects. During incubation, the moisture level of vermiculite was checked weekly and water was added when necessary. Both vermiculite and water were autoclaved prior to use to minimize microbial contamination. Eggs were discarded if fungal growth was observed. Post oviposition, females were released at their initial capture locations. A total of 56 females laid eggs during the study, of which 43 produced at least one successfully hatched offspring (Table 1). Clutch size ranged from 1 to 12 eggs per female. Most females (*n*=40 of 56; 71.4%) produced clutches containing 8–12 eggs.

**Table 1.** Effects of incubation temperature on embryonic development, physiology and hatchling traits in *Psammophilus dorsalis*

	Temperature treatment				
	25°C	30°C	35°C	F2 (25–30°C)	F3 (25–30–35°C)
No. eggs (no. clutches)	119 (47)	133 (55)	73 (32)	72 (23)	75 (32)
No. eggs hatched	77	81	0	46	33
Hatching success (%)	64.7	60.9	0	63.9	44
Development time (days)	61.84±0.24	43.60±0.19	NA	48.76±0.15	46.24±0.20
HR all eggs (bpm)	87.60±1.52	90.57±1.29	105.18±3.58	93.79±2.41	88.83±2.06
HR hatched eggs (bpm)	79.76±0.59	86.92±0.87	NA	83.09±1.14	88.43±1.08
HR slope for all eggs	0.02±0.06	0.12±0.10	1.03±0.16	0.07±0.10	0.01±0.14
HR slope for hatched eggs	0.12±0.04	0.31±0.06	NA	0.17±0.06	−0.04±0.10
Final mass for hatched eggs (g)	2.15±0.04	2.01±0.05	NA	1.98±0.04	2.23±0.05
Mass slope for all eggs	0.03±0.00	0.03±0.00	0.02±0.00	0.03±0.00	0.03±0.00
Mass slope for hatched eggs	0.03±0.00	0.04±0.00	NA	0.03±0.00	0.04±0.00
Hatchling SVL (cm) (no. hatchlings)	2.82±0.04 (34)	2.72±0.03 (53)	NA (0)	2.72±0.03 (21)	2.72±0.02 (23)
Hatchling mass (g) (no. hatchlings)	0.83±0.02 (34)	0.78±0.02 (53)	NA (0)	0.80±0.02 (21)	0.72±0.02 (23)

Eggs were incubated under constant (25°C, 30°C, 35°C) and fluctuating regimes (F2: 25–30°C; F3: 25–30–35°C). Values are means±s.e.m. Hatching success (%) is based on the number of eggs hatched per treatment. Development time is shown for successfully hatched eggs only. Heart rate (HR) is presented for all eggs and for hatched eggs separately. Slopes represent rates of change in heart rate (bpm day^−1^) and egg mass (g day^−1^) across development time (egg age). Hatchling traits [final egg mass, snout to vent length (SVL) and body mass] were measured within 7 days of hatching. No eggs hatched at 35°C.

Ethics clearance and protocols for this project have been approved by the institute animal ethics committee (CAF/Ethics/995/2023). Collection permits for *P. dorsalis* was not required as this species is not protected under the Schedules of the Indian Wildlife (Protection) Act, 1972.

### Incubation treatments

Nest temperatures in the field (*n*=6) were recorded using Thermochron iButtons (Maxim Integrated DS1921G) placed in the ground at a depth of 8 cm, corresponding to typical nest depth for *P. dorsalis* (Balakrishna and Achari, 2014). Based on these field measurements (Fig. S1), five incubation treatments were designed. Three constant temperature treatments were 25°C, 30°C and 35°C. The 25°C treatment represented the average nest temperature observed in the field and 35°C represented typical daytime peak temperatures in sun exposed nests. Two fluctuating temperature regimes were also selected to incorporate thermal variation. In the F2 treatment, temperature alternated every 24 h between 25°C and 30°C (mean overall temperature=27.5°C). In the F3 treatment, temperature alternated every 24 h among 25°C, 30°C and 35°C in cyclical order (mean=30°C). Comparison between the constant 30°C and fluctuating F3 treatment allowed separation of mean temperature effects from temperature variability. Unlike F3, F2 did not expose embryos to potentially stressful temperatures but instead imposed moderate thermal variation within the range commonly observed across nesting sites. This treatment allowed us to assess whether moderate temperature fluctuations influence embryonic development and survival in the absence of extreme thermal events. Eggs were incubated in programmable incubators (PHCBI MIR-154) maintained at assigned temperature regimes until hatching or developmental failure.

### Egg and hatchling measurements

Embryonic heart rate was measured weekly throughout development using a digital egg monitor (Egg Buddy, Avitronics, Cornwall, UK), which detects embryonic heartbeat through the eggshell without disturbing the embryo. Eggs were removed from their respective incubator and embryonic heart rate was measured within 1 min in a room maintained at 28°C. Multiple readings were taken from different positions of the egg and averaged. Egg mass (in g) was also measured weekly using a portable digital balance (accuracy up to 0.01 g). Eggs were classified as either successfully hatched (1) or failed to hatch (0). Hatching success for each treatment was calculated as the proportion of eggs that hatched over total the number of eggs in a temperature treatment. For eggs that hatched, we calculated developmental time as the number of days from oviposition to hatching. Finally, snout to vent length (SVL in mm using a ruler) and body mass (using a digital scale with accuracy up to 0.01 g) were measured for all hatchlings within 7 days of emergence.

### Statistical analyses

Embryonic heart rate and egg mass were analysed separately using linear mixed-effects models (LMMs), with egg age (days), incubation treatment and their interaction as fixed effects. Egg identity was included as a random intercept to account for repeated measurements of the same egg. For heart rate, linear and quadratic models were compared; inclusion of a quadratic term did not improve model fit (ΔAIC=43), and therefore linear models were retained. Analyses were conducted for all eggs and repeated using only eggs that successfully hatched. Hatching success was analysed using a generalized linear mixed-effects model (GLMM) with a binomial error distribution and logit link function, that included incubation treatment as a fixed effect and maternal identity as a random intercept. Developmental time (days from oviposition to hatching) was analysed using an LMM with incubation treatment as a fixed effect and maternal identity as a random intercept. Hatchling SVL and body mass were similarly analysed using LMMs with incubation treatment as a fixed effect and maternal identity as a random intercept. Model assumptions for linear mixed-effects models were assessed by visual inspection of residual and *Q*–*Q* plots to verify homogeneity of variance and normality of residuals. For binomial GLMMs, model fit and overdispersion were evaluated using DHARMa package (https://CRAN.R-project.org/package=DHARMa). Models were fitted using the lmer or glmer functions in the lme4 package (https://CRAN.R-project.org/package=lme4) in R, followed by Type III ANOVA, and *post hoc* comparisons using Tukey-adjusted estimated marginal means in the emmeans (https://CRAN.R-project.org/package=emmeans) package when relevant.

## RESULTS AND DISCUSSION

### Effects of incubation temperature on embryonic heart rate

**Fig. 1.**
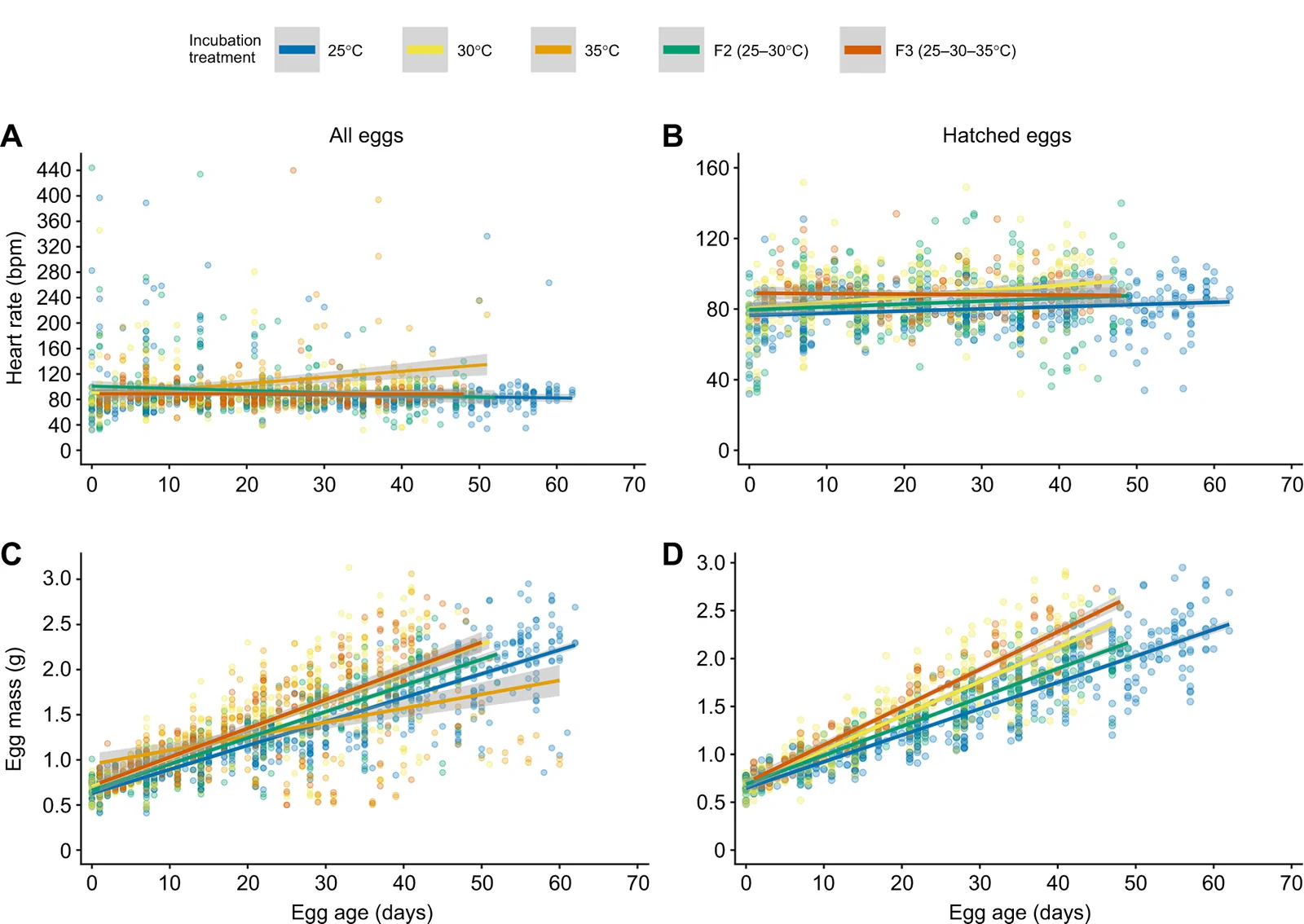
**Effects of incubation treatment on embryonic heart rate and egg mass across development (egg age) in *Psammophilus dorsalis*.** (A) Heart rate trajectories for all eggs across incubation treatments. (B) Heart rate trajectories for eggs that successfully hatched. (C) Egg mass trajectories for all eggs. (D) Egg mass trajectories for eggs that successfully hatched. Eggs were incubated under constant (25°C, 30°C, 35°C) and fluctuating regimes (F2, 25–30°C; F3, 25–30–35°C). Points represent individual measurements and lines show linear model fits ±s.e.m. Note that *y*-axis scales differ between panels. See Table 1 for sample sizes.

Incubation treatment significantly affected embryonic heart rate during development (egg age×temperature interaction: *F*_4_,_1288_=8.92, *P*<0.001). Heart rate increased with embryonic age most rapidly under the constant 35°C treatment (slope=1.025±0.160 bpm day^−1^; Fig. 1A, Table 1), whereas slopes were similar for 25°C, 30°C, F2 and F3 treatments (all pairwise *P*≥0.89). Among embryos that successfully hatched, heart rate trajectories across embryonic age also differed among treatments (egg age×temperature interaction: *F*_3_,_1053_=3.90, *P*=0.009; Fig. 1B, Table 1). Embryos incubated at 30°C exhibited the steepest increase in heart rate across development (slope=0.314±0.061 bpm day^−1^), whereas increases were smaller at 25°C and F2 (0.116 and 0.166 bpm day^−1^, respectively) and negligible in F3 (−0.037±0.097 bpm day^−1^). Pairwise comparisons indicated that the slope at 30°C was significantly higher than those at 25°C (*P*=0.033) and F3 (*P*=0.012), whereas the remaining comparisons were non-significant (*P*≥0.30).

Extremely high and variable heart rates were common in the full dataset (Fig. 1A) but largely absent among embryos that survived to hatching (Fig. 1B), suggesting that embryos experiencing elevated heart rates were less likely to survive. Consistent with this, mean heart rate was highest at 35°C (105.18±3.58 bpm; Table 1) where no embryos survived. As embryonic heart rate is closely linked to metabolic rate (Du et al., 2009, 2011), elevated heart rate indicates heightened metabolic demand, likely reflecting thermal stress for developing embryos. Similar patterns have been reported in reptiles, where elevated metabolic rates under thermal stress can exceed physiological limits and reduce survival (Angilletta et al., 2013; Hall and Warner, 2020). Egg identity explained large variation (67%), highlighting intrinsic (likely maternal) effects on embryonic physiology (Warner et al., 2007).

### Effects of incubation temperature on egg mass

Egg mass differed significantly among temperature treatments over time (egg age×temperature: *F*_4_,_1585_=43.60, *P*<0.001; Fig. 1C). Eggs at 35°C showed the slowest increase in mass during development, whereas eggs at 30°C and F3 exhibited the fastest increase. However, among successfully hatched embryos (Fig. 1D), egg mass increased similarly across treatments despite differences in survival. Notably, surviving embryos in the F3 treatment exhibited the steepest increase in mass (slope=0.040±0.001 g day^−1^), despite relatively low hatching success and hatchling mass (Table 1).

**Fig. 2.**
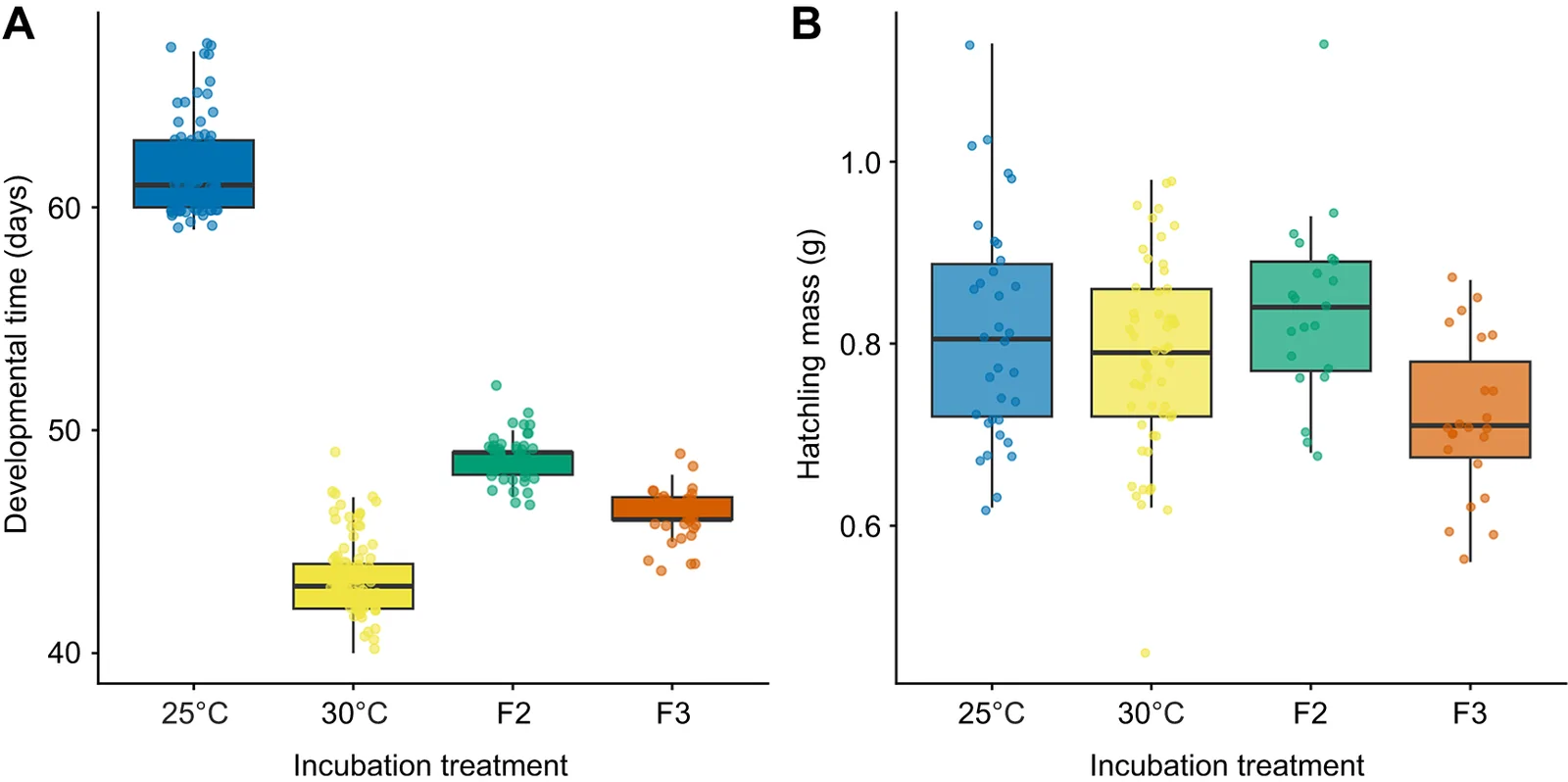
**Effects of incubation treatment on developmental time and hatchling body mass.** (A) Developmental time of embryos incubated under constant (25°C, 30°C, 35°C) and fluctuating regimes (F2, 25–30°C; F3, 25–30–35°C). (B) Hatchling body mass. Boxes show medians and interquartile ranges, whiskers extend to 1.5×IQR and points represent individual observations. See Table 1 for sample sizes.

Unlike heart rate, changes in egg mass during development were broadly similar between all eggs (Fig. 2A) and the subset of hatched eggs (Fig. 2B). This suggests that egg mass alone is a poor predictor of survival, as eggs can increase in mass over time and still fail to hatch. Reptile eggs typically gain mass through water uptake (Booth, 2006) and faster mass gain under warmer or fluctuating conditions may reflect increased metabolic activity and altered hydric exchange (De-Lima et al., 2022). However, these results indicate that an increase in mass does not necessarily translate into successful development.

### Effects of incubation temperature on hatching success and developmental time

Hatching success differed significantly among incubation treatments (likelihood ratio test: $\chi_{4}^{2}$=130.29, d.f.=4, *P*<0.001). Hatching success was highest under 25°C (64.7%), followed by F2 (63.9%) and 30°C (60.9%), but declined under F3 (44.0%), with complete failure at 35°C (0%; Table 1). The reduced hatching success under F3 and complete mortality at 35°C are consistent with thermal death time (TDT) theory, which predicts that survival depends on both the intensity and duration of heat exposure (Rezende et al., 2014; Jørgensen et al., 2021; Ørsted et al., 2024). Embryos may continue to increase in size, like those in the 35°C treatment, while simultaneously accumulating physiological damage that ultimately compromises survival. Elevated heart rates of embryos in the 35°C treatment probably reflect this increasing physiological stress and metabolic demand. The complete failure at 35°C indicates that embryos of *P. dorsalis* accumulated lethal levels of thermal damage under sustained exposure to high temperatures. Similarly, the reduced hatching success in F3 indicates that repeated exposure to high thermal spikes can also generate sufficient cumulative damage to impair survival, despite periods of cooler temperatures between exposures. These findings support growing evidence that both mean temperature and thermal variability influence embryonic survival by determining the rate at which thermal damage accumulates (Noble et al., 2018a; Raynal et al., 2022). In contrast, the relatively high hatching success observed under 25°C, 30°C and F2 conditions suggests that thermal conditions remained within a range that limited damage accumulation and allowed successful development. Suitable temperatures for development are, however, species specific as the closely related *C. versicolor* showed hatching success at a constant 35°C (Radder et al., 2002).

For eggs that survived to hatchling, developmental time was shortened by higher temperatures (*F*_3_,_197_=4961.1, *P*<0.001, Table 1). Eggs incubated at 25°C took the longest to hatch (61.84±0.24 days), whereas those at 30°C developed fastest (43.60±0.19 days), with F2 (48.76±0.15 days) and F3 (46.24±0.20 days) resulting in intermediate developmental times (all pairwise comparisons: *P*<0.001; Fig. 2A, Table 1). Accelerated development at higher temperatures is a widespread pattern in reptiles (Noble et al., 2018a). Although developmental times were similar in treatments that were constant or variable, survival outcomes differed. Eggs exposed intermittently to 35°C (in F3) had a higher risk of mortality than those exposed to the same mean temperature conditions (constant 30°C). Thus, intermittent exposure to high temperature conditions can result in accumulated physiological damage that is lethal (Rezende et al., 2014).

Maternal identity explained substantial variation in hatchling success (variance=67%) and developmental time (variance=75%), indicating strong maternal effects on embryo development and survival (Duchak and Burke, 2022). Maternal investment in eggs, particularly variation in egg provisioning and maternal corticosterone levels, can affect hatching success and offspring phenotype independent of incubation conditions (Leibold et al., 2026). The substantial maternal effect observed here suggests that differences among females in egg quality or physiological state may contribute strongly to variation in developmental outcomes. It is currently unknown whether sex determination is temperature dependent in this species, and because sex could not be determined at the hatchling stage, potential effects of incubation temperature on offspring sex ratios could not be assessed.

### Effects of incubation temperature on hatchling size

Hatchling mass (*F*_3_,_113.8_=4.60, *P*=0.004) but not SVL (*F*_3_,_102.9_=2.19, *P*=0.090) differed among temperature treatments. Hatchlings from the F3 treatment were significantly lighter than those incubated at 25°C (*post hoc P*=0.002; Fig. 2B, Table 1), whereas other pairwise comparisons were not significantly different. Reduced hatchling mass under F3 contrasts with studies reporting larger hatchlings under fluctuating temperatures (Medina et al., 2025) but aligns with the interpretation that greater thermal variation can impose energetic costs (Booth, 2006). Although hatchlings from F3 were approximately 8% lighter than those from the corresponding constant-temperature treatment (30°C), this difference was not statistically significant (Stocker et al., 2024). Embryos developing under warmer and more variable regimes probably experience elevated metabolic expenditure and transient oxygen limitation, thereby reducing the efficiency of yolk-to-tissue conversion, resulting in lower hatchling body mass (Booth, 2006; Raynal et al., 2022). Cooler conditions appear to allow more efficient yolk absorption over longer developmental periods, producing heavier neonates (Booth, 2000, 2006). Such carry-over effects on hatchling quality can have important fitness consequences (While et al., 2018).

### Conclusion

Overall, our results show that thermal variability, particularly regimes that include high-temperature peaks, imposes substantial physiological costs during development. Although moderate fluctuations (F2) were relatively benign, exposure to higher variable temperatures (F3) reduced survival and hatchling mass, despite similar developmental rates. Embryos can develop rapidly under warmer conditions but they still experience reduced survival and offspring quality. Changes in embryonic heart rate and egg mass over time indicate that mortality was not simply associated with reduced egg mass gain during development. Instead, elevated heart rates under warmer treatments probably reflect increased physiological stress and metabolic demands associated with thermal exposure, potentially contributing to reduced survival.

In natural nesting environments, *P. dorsalis* already experiences temperatures exceeding 35°C. However, if the frequency or duration of such high-temperature events increases under climate warming, embryonic survival could decline sharply. While the F3 regime represents an experimental rather than a natural scenario, our results suggest that frequent exposure to high temperatures may impose developmental costs. The magnitude of these effects in natural populations will depend on the buffering effects of nest thermal environments and the capacity of females to adjust egg laying behaviour. Females may reduce embryonic exposure to extreme temperatures by shifting egg laying phenology or selecting cooler nesting sites. Finally, differences between *P. dorsalis* and *C. versicolor* (Radder et al., 2002; Ben et al., 2026) indicate that even similarly sized and co-occurring species may differ in sensitivity to thermal extremes during development, which highlights the need for species-specific assessments of climate vulnerability under more realistic thermal regimes.

## Supplementary Material

10.1242/jexbio.252800_sup1 Supplementary information
