# Supplementary material for: Thermal fluctuations affect embryonic physiology in a tropical agamid lizard
Source: J Exp Biol. 2026 Aug 11;229(15):jeb252800. doi: 10.1242/jeb.252800 (PMC7619429; doi:10.1242/jeb.252800)
Supplement: Supplementary file 1 [file jexbio-229-252800-s1.pdf]

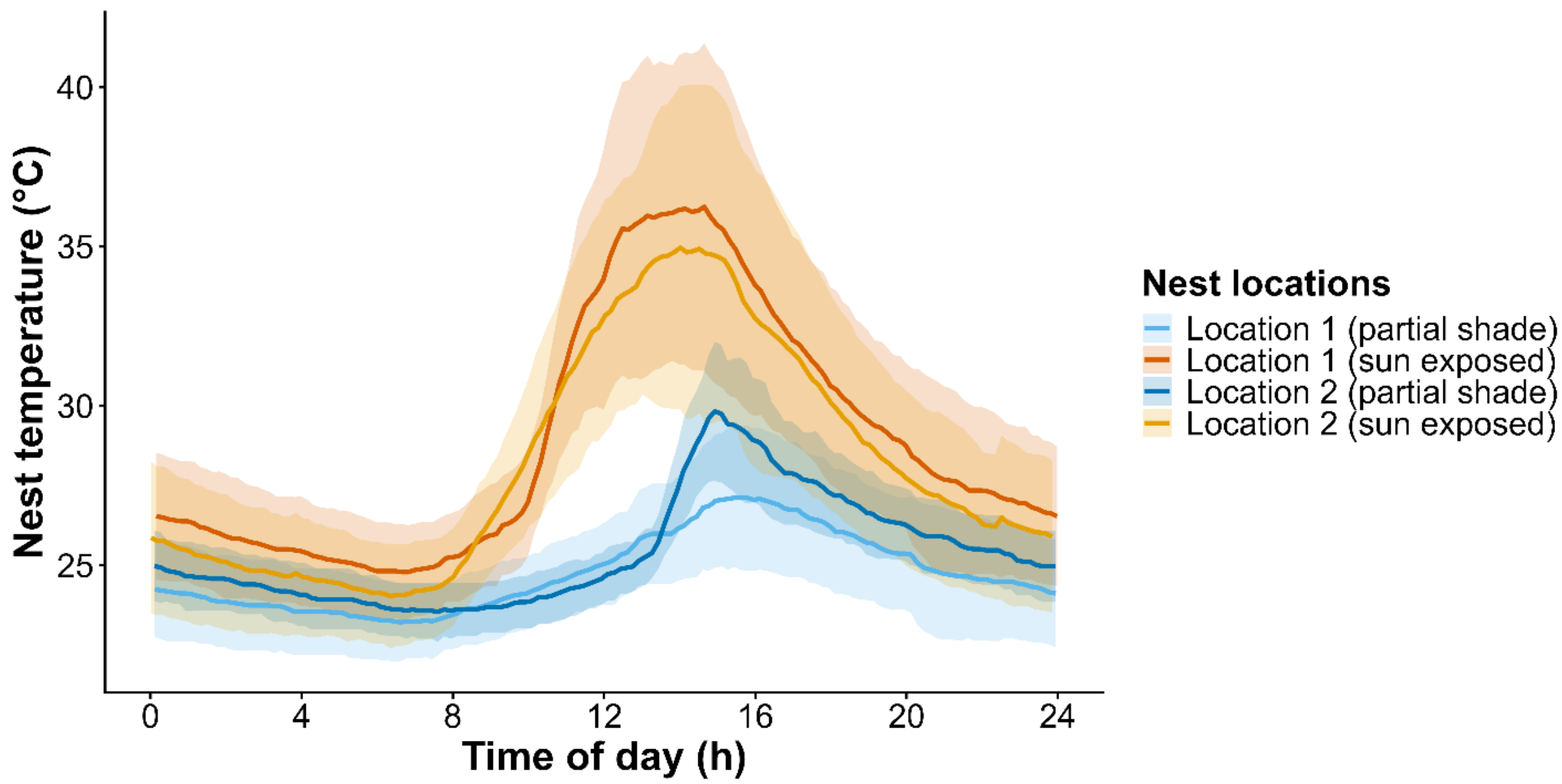

Fig. S1. Diurnal variation in nest temperature across four potential nesting locations of *Psammophilus dorsalis*. Temperature was recorded using iButton data loggers buried at a depth of 8 cm, corresponding to the typical nest depth. Location 1 and Location 2, separated by 28 km, each contained one partially shaded and one sun-exposed nesting site. Lines represent mean nest temperatures over a 24-h period, and shaded regions indicate variability (± SD) among days.

Journal of Experimental Biology • Supplementary information
